# Cardiac changes during the peri‐menopausal period in a VCD‐induced murine model of ovarian failure

**DOI:** 10.1111/apha.13290

**Published:** 2019-05-31

**Authors:** Rosephine Del Fernandes, Alexandra Hall, Melissa Ferguson, Ilka Lorenzen‐Schmidt, Vishali Balasubramaniam, W. Glen Pyle

**Affiliations:** ^1^ Department of Biomedical Sciences University of Guelph Guelph Ontario Canada

**Keywords:** oestrogen receptors, heart, myofilaments, peri‐menopause

## Abstract

**Aim:**

Cardiovascular disease (CVD) risk is lower in pre‐menopausal females vs age matched males. After menopause risk equals or exceeds that of males. CVD protection of pre‐menopausal females is ascribed to high circulating oestrogen levels. Despite experimental evidence that oestrogen are cardioprotective, oestrogen replacement therapy trials have not shown clear benefits. One hypothesis to explain the discrepancy proposed hearts remodel during peri‐menopause. Peri‐menopasual myocardial changes have never been investigated, nor has the ability of oestrogen to regulate heart function during peri‐menopause.

**Methods:**

We injected female mice with 4‐vinylcyclohexene diepoxide (VCD, 160 mg/kg/d IP) to cause gradual ovarian failure over 120d and act as a peri‐menopausal model

**Results:**

Left ventricular function assessed by Langendorff perfusion found no changes in VCD‐injected mice at 60 or 120 days compared to intact mice. Cardiac myofilament activity was altered at 60 and 120 days indicating a molecular remodelling in peri‐menopause. Myocardial TGF‐β1 increased at 60 days post‐VCD treatment along with reduced Akt phosphorylation. Acute activation of oestrogen receptor‐α (ERα) or ‐β (ERβ) depressed left ventricular contractility in hearts from intact mice. ER‐regulation of myocardial and myofilament function, and myofilament phosphorylation, were disrupted in the peri‐menopausal model. Disruption occurred without alterations in total ERα or ERβ expression.

**Conclusions:**

This is the first study to demonstrate remodelling of the heart in a model of peri‐menopause, along with a disruption in ER‐dependent regulation of the heart. These data indicate that oestrogen replacement therapy initiated after menopause affects a heart that is profoundly different from that found in reproductively intact animals.

## 1 INTRODUCTION

Cardiovascular disease (CVD) risk rises in both sexes with ageing but women exhibit an interesting and well‐known phenomenon in which CVD risk for pre‐menopausal women is lower than age‐matched males, but equal or higher than men after menopause. The correlation between ovarian oestrogen production and CVD led to the development of the “Oestrogen Protection Hypothesis”. Despite initial experimental and clinical studies supporting a cardioprotective role for oestrogen,^1^, ^2^, ^3^ the evidence for oestrogen replacement therapy is confounding.^4^

Most research that sought to determine how the heart changes after menopause used ovariectomized (OVX) animals for experimental subjects. While OVX produces an oestrogen‐deficient state similar to that seen in post‐menopausal women, the sudden onset and loss of all ovarian function does not recapitulate the gradual onset, prolonged transitory phase and retention of ovarian tissue (and androgen secretion) that occurs in menopause for most women. The inability to replicate these physiological elements of menopause in experimental animals limits the applicability of this research to the human population. Understanding the physiological changes that occur in the hearts of women during and after menopause is crucial to determining why the rates of CVD rise dramatically and outcomes worsen in post‐menopausal women.

Rodents experience irregular cyclicity around 9‐12 months of age, but not the very low oestrogen levels that occur in humans,^5^ which makes ageing rodents an unsuitable model for menopause. Hoyer and colleagues created a model of menopause in which ovarian function is gradually reduced following a series of injections with 4‐vinylcyclohexene diepoxide (VCD).^6^ Mice enter menopause ~120 days after VCD injections and the gradual transition into a menopausal state mimics the physiological changes to the hormonal milieu that characterize the peri‐menopausal transition to menopause.^6^, ^7^ The impact of VCD is confined to ovarian toxicity and no other organ system is impacted by the chemical.^6^ The advantages of this model of menopause include gradual loss of oestrogens; retention of ovarian tissue to maintain some steroidogenesis; and the ability to use young or old animals to control for the effects of ageing.^5^, ^6^ This new model of menopause offers a tremendous opportunity to investigate the cardiac changes that occur with menopause, including regulation by oestrogen receptors and changes in the molecular elements of heart function. This study sought to identify changes in the molecular basis of heart function and regulation by oestrogen receptors during the transition to a menopausal state. We found that while myocardial function remains unaffected by the transition to menopause, myofilament function and oestrogen receptor regulation of the heart are significantly affected. This is the first study to identify critical changes in cardiac physiology and regulation during the peri‐menopausal transition to a post‐menopausal phase.

## 2 MATERIALS AND METHODS

### 2.1 Animals

Sexually mature CD1 female mice aged 78‐105 days were obtained from Charles River Laboratories (St. Constant, QC). Mice were housed in groups of four at the Central Animal Facility at the University of Guelph on a 12‐h light/dark cycle. Food and water were provided ad libitum. Animals were allowed to acclimatize to the animal facility for at least one week prior to the initiation of treatment. All procedures conducted were in accordance with the guidelines set by the Animal Care and Use Committee of the University of Guelph and the Canadian Council on Animal Care.

### 2.2 VCD mouse model of peri‐menopause

Mice were weighed and given daily intraperitoneal injections (160 mg/kg) of VCD (MilliporeSigma, Oakville, ON) for 15 consecutive days as per the protocol of Perez et al^8^. VCD accelerates atresia by inducing the selective loss of primary and primordial follicles in the ovary but leaves intact the rest of the ovary for residual hormone production. VCD mediates its ovarian effects by inhibiting autophosphorylation of membrane receptor c‐kit.^9^ Control mice were injected with vehicle (sesame oil; intact). Vaginal cytology was used to confirm lack of estrus cycles indicating ovarian failure.^10^, ^11^ Mice were considered acyclic after 10 consecutive days of persistent diestrus. Mice showed disrupted estrus cycles (prolonged to ~6 days) by day 60 (VCD 60 D) and acyclicity by day 120 (VCD 120 D). Day 60 was chosen as the mid‐point of the peri‐menopausal period and day 120 represents the end of peri‐menopause and the onset of ovarian failure.

### 2.3 Langendorff perfusion

Excised mouse hearts were perfused using a Langendorff apparatus as described in Yang and Pyle.^12^ Hearts werepaced at ~425 bpm and perfused for 15 minutes prior to any intervention to establish baseline function. Baseline values were taken as an average of the last 30 seconds before treatment. Hearts were then treated for 15 minutes with 100 nM 4,4′,4″‐(4‐propyl‐[1H]‐pyrazole‐1,3,5‐triyl)trisphenol (PPT, oestrogen receptor‐α agonist), 1 nM diarylpropionitrile (DPN, oestrogen receptor‐β agonist), or ethanol (control) vehicle. After perfusion hearts were snap frozen in liquid nitrogen and stored at −80°C.

### 2.4 Myofilament isolation

Cardiac myofilaments were isolated according to the protocol of Yang and Pyle.^12^ Briefly, hearts were homogenized in ice cold Standard Buffer (60 mM KCl, 30 mM Imidazole (pH 7.0), 2 mM MgCl_2_) containing protease and phosphatase inhibitors. The homogenate was centrifuged at 12,000 *g* for 15 minutes at 4°C and the pellet was resuspended for 45 minutes in ice cold Standard Buffer plus 1% Triton X‐100. This mixture was centrifuged at 1,100 *g* for 15 minutes at 4°C and the pellet washed three times in ice‐cold Standard Buffer. Protein concentration was measured with a Bio‐Rad Bradford Protein Assay (Bio‐Rad Laboratories Ltd., Mississauga, ON).

### 2.5 Actomyosin MgATPase assay

Actomyosin MgATPase activity was determined using a modified Carter assay.^12^ Isolated cardiac myofilaments (25 μg) were incubated in reaction buffers which contained varying levels of free calcium. Buffers containing calcium concentrations of pCa 4.0 (activating) and pCa 9.0 (relaxing) were mixed to prepare the reaction buffers used. Free calcium was calculated using the program described in Patton et al^13^. Myofilaments were incubated in reaction buffers for 10 minutes at 32°C and the reactions were quenched with ice cold 10% trichloroacetic acid. The production of inorganic phosphate was measured by adding equal volumes of 0.5% FeSO_4_ and 0.5% ammonium molybdate in 0.5 M H_2_SO_4_.

### 2.6 Myofilament protein phosphorylation

Myofilament proteins (10 μg) were separated using 12% SDS‐PAGE and fixed in 50% methanol‐10% acetic acid at room temperature overnight. Total protein phosphorylation was determined by staining gels with Pro‐Q Diamond phosphoprotein stain (Thermo Fisher Scientific, Mississauga, ON) according to the manufacturer's instructions. Gels were imaged using a Bio‐Rad ChemiDoc MP Imaging System (Bio‐Rad Laboratories Ltd., Mississauga, ON) and data were analysed with ImageJ (NIH, Bethesda, MD). Total protein load was determined by Coomassie staining of the same gels and protein phosphorylation was normalized to total protein.

### 2.7 Immunoblotting

Immunoblotting was conducted using the protocol from Yang and Pyle.^12^ Myocardium was homogenized in ice‐cold standard buffer and protein quantified using a Bio‐Rad Bradford Protein Assay. Whole heart homogenates (50 μg for Akt blots and 100 μg for all others) were resolved by SDS‐PAGE using 10% (Akt), 12% (oestrogen receptors), or 15% (TGF‐β1 and TNF‐α) separating gels. Proteins were transferred to nitrocellulose membranes (100 V, 3 h, room temperature) and probed with primary antibodies overnight at 4°C. Antibodies for oestrogen receptor‐α (ERα, sc‐542),^14^ oestrogen receptor‐β (ERβ, sc‐8974),^14^, ^15^ TNF‐α (sc‐1350),^16^ and TGF‐β1 (sc‐146)^17^ (Santa Cruz Biotechnology, Dallas, TX) were diluted 1:1000 in TBS with 1% dry milk powder. Total Akt (#4691)^18^, ^19^ and phosphorylated Akt (#4060)^18^, ^19^ (Cell Signaling Technology, Danvers, MA) were incubated under the same conditions except at a dilution of 1:2,000. Antibody for α‐actinin (MAB1682, MilliporeSigma, Oakville, ON)^20^ was diluted 1:2,000 and incubated for 1 hour at room temperature. Secondary antibodies conjugated to horseradish peroxidase were used at a dilution of 1:5,000 and added for 1 hour at room temperature (MilliporeSigma, Oakville, ON). Protein bands were detected using Western Lightning (PerkinElmer Life and Analytical Sciences, Woodbridge, ON) and Bio‐Rad ChemiDoc MP Imaging System (Bio‐Rad Laboratories Ltd., Mississauga, ON). Data were analysed with ImageJ (NIH, Bethesda, MD).

### 2.8 Statistical analysis

All data are shown as mean ± SEM. Statistical analysis was done using two‐way ANOVA and a post hoc Tukey's Test. *P* < 0.05 was considered statistically significant.

## 3 RESULTS

### 3.1 Cardiac changes during the peri‐menopausal transition to a menopausal state

**Figure 1:**
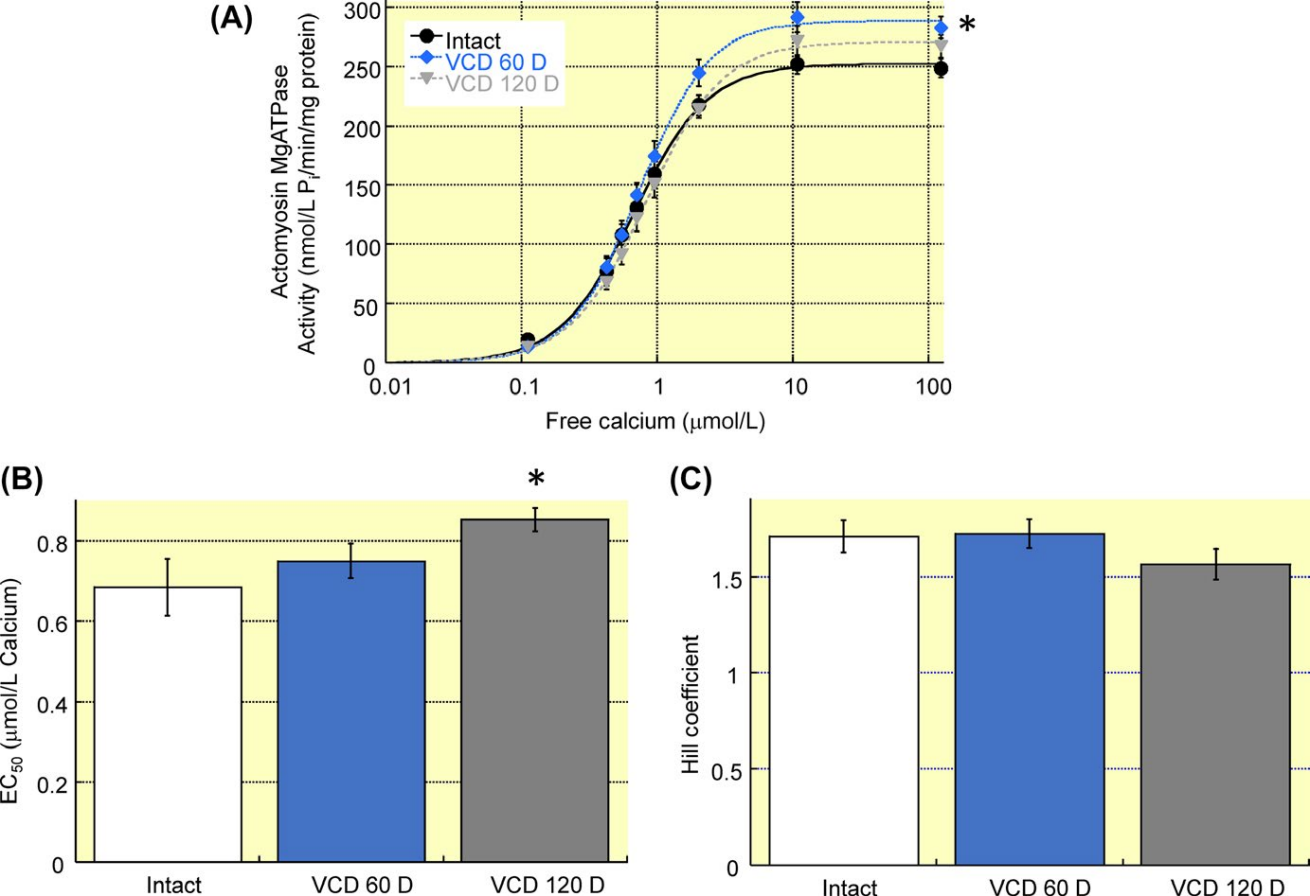
Cardiac myofilament function in a mouse model of peri‐menopause. A, Cardiac actomyosin MgATPase activity was measured across a range of free calcium concentrations. Maximum activity increased at day 60 post‐VCD treatment as compared to intact mice. B, At day 120 post‐VCD treatment EC_50_ was significantly higher in VCD‐treated mice as compared to intact controls. C, Hill coefficient was not affected at either VCD timepoint. Note: VCD 60 D, 60 days after VCD injections; VCD 120 D, 120 days after VCD injections. N = 7 in each group. *P < 0.05 vs intact control

**Figure 2:**
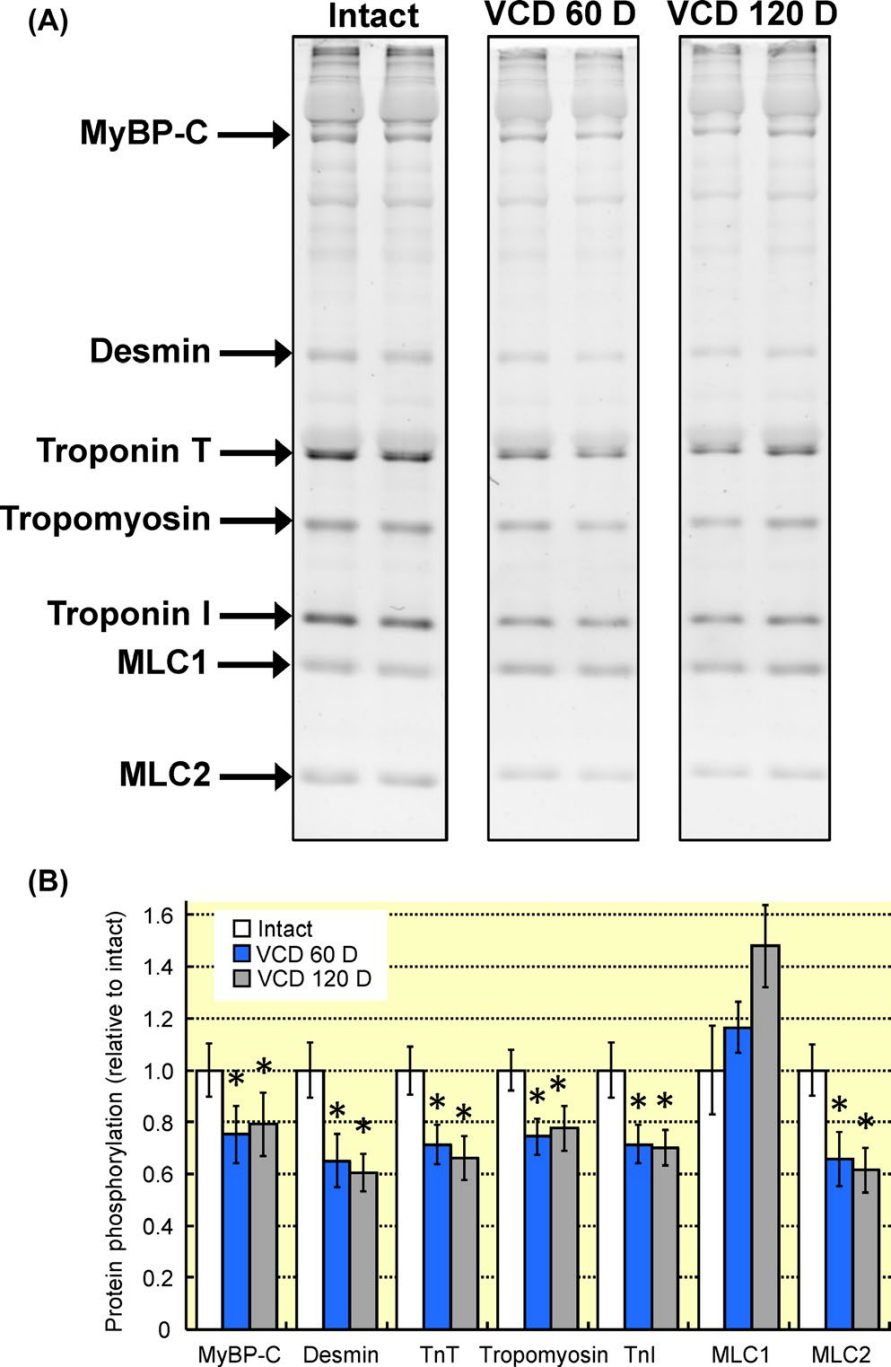
Changes in cardiac myofilament protein phosphorylation associated with ovarian failure. A, Cardiac myofilaments were separated by SDS‐PAGE and phosphorylated proteins were stained with ProQ‐Diamond. B, Cardiac myofilament protein phosphorylation was significantly reduced by day 60 post‐VCD treatment as compared to intact controls with the exception of MLC1. The reduction in protein phosphorylation remained at day 120 post‐VCD treatment. Note: MyBP‐C, myosin binding protein C; TnT, troponin T; TnI, troponin I, MLC1, myosin light chain 1; MLC2, myosin light chain 2; VCD 60 D, 60 days after VCD injections; VCD 120 D, 120 days after VCD injections. N = 7 per group. *P < 0.05 vs intact control

Left ventricular function was not different between hearts from intact mice or VCD‐treated mice (60 or 120 days post‐treatment) (Table 1). Cardiac myofilament function was altered by day 60 post‐VCD treatment as demonstrated by the increase in maximum actomyosin MgATPase activity from 248 ± 8 nM P_i_/min/mg protein to 283 ± 9 P_i_/min/mg protein (Figure 1). At day 120 post‐VCD treatment myofilament calcium sensitivity—as represented by an increase in EC_50_ from 0.68 ± 0.07 μM to 0.84 ± 0.03 μM—decreased significantly compared to intact mice (Figure 1B). Cardiac myofilament protein phosphorylation was generally decreased in VCD‐treated mice (Figure 2). By day 60 post‐VCD treatment, myosin binding protein C, desmin, troponin T, tropomyosin, troponin I and myosin light chain 2 all showed reduced phosphorylation levels relative to intact controls. These changes remained significant at day 120 post‐VCD treatment. Together these data show that left ventricular function is not altered in a mouse model of peri‐menopause, but the underlying molecular mechanisms that drive function are impacted by gradual ovarian failure.

**Table 1:** Baseline functional values

Group	LVESP (mm Hg)	LVEDP (mm Hg)	d*P*/d*t*_max_ (mm Hg/s)	d*P*/d*t*_min_ (mm Hg/s)
Intact	90.8 ± 1.6	2.6 ± 0.4	2990 ± 55	−2300 ± 56
VCD 60 D	90.0 ± 1.8	3.7 ± 0.3	2897 ± 48	−2238 ± 49
VCD 120 D	90.5 ± 1.2	3.1 ± 0.3	2967 ± 48	−2253 ± 46

Note:

d*P*/d*t*_max_, peak rate of contraction; d*P*/d*t*_min_, peak rate of relaxation; LVEDP, left ventricular end diastolic pressure; LVESP, left ventricular end‐systolic pressure. N = 7 per group.

### 3.2 Myocardial cytokines are altered in peri‐menopause

**Figure 3:**
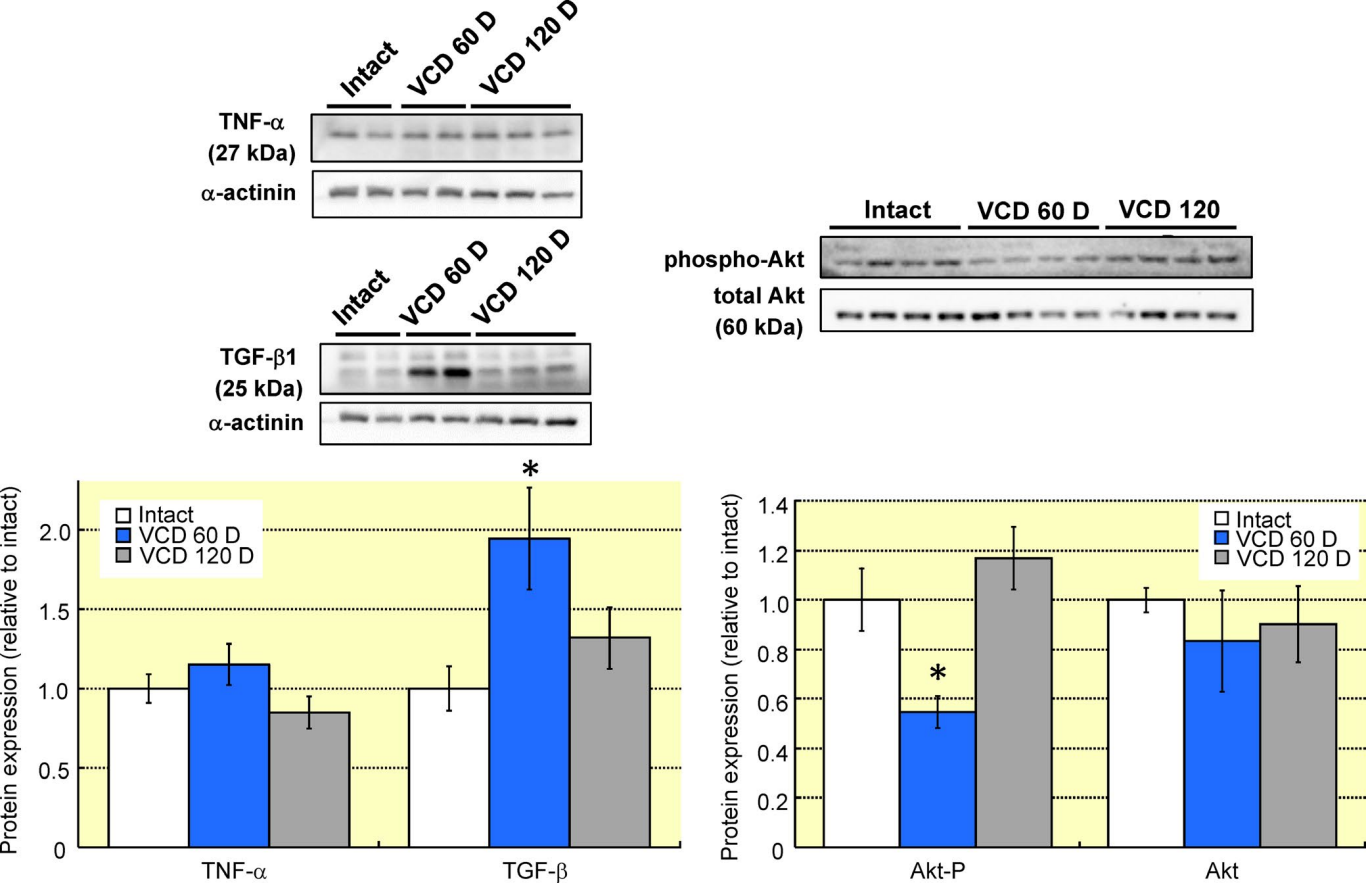
Ovarian failure alters myocardial cytokines and Akt activation. A, Myocardial protein levels of TNF‐α and TGF‐β1 were assessed with immunoblotting. Ovarian failure did not alter TNF‐α but TGF‐β1 was increased by 94.3 ± 32.0% during peri‐menopause. B, Akt phosphorylation decreased by 46.3 ± 6.5% at 60 D VCD but returned to the same levels as intact myocardium by 120 D VCD. Total Akt protein levels did not change with VCD injection. N = 4 per group. *P < 0.05 vs intact control

Menopause and peri‐menopause are characterized by alterations in serum cytokines.^21^, ^22^ To determine if myocardial cytokines are similarly affected we measured myocardial TNF‐α and TGF‐β1 by immunoblotting. Myocardial TGF‐β1 increased ~twofold at day 60 but was not significantly different than intact controls by day 120 post‐VCD (Figure 3). TNF‐α was not significantly different at either timepoint. Decreased 17β‐estradiol^23^ and TGF‐β1^24^ both decrease Akt phosphorylation which could explain the reduction in myofilament phosphorylation and trend towards a decrease in myofilament calcium sensitivity. Myocardial Akt phosphorylation was reduced by 46.3% on day 60 post‐VCD and not significantly changed on day 120. Total Akt levels did not significantly differ at any timepoint. The decline in Akt activation coincides with decreased myofilament phosphorylation on day 60 but is unable to account for myofilament differences on day 120, suggesting a complex network of signalling molecule changes during the transition to menopause.

### 3.3 Effects of acute oestrogen receptor activation on left ventricular function

**Figure 4:**
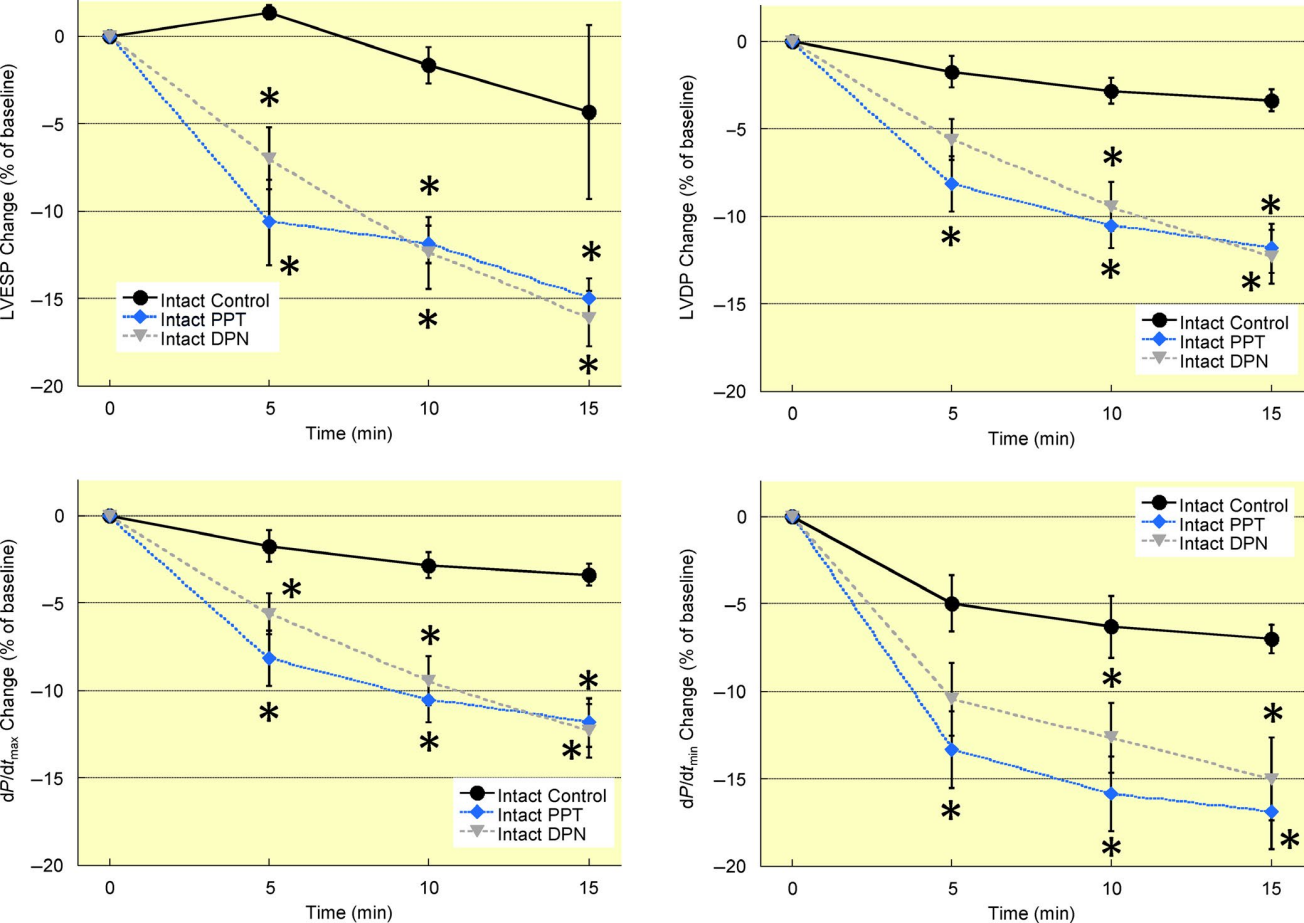
Acute oestrogen receptor activation decreases left ventricular contractility in intact mice. Hearts from intact mice were perfused for 15 min with agonists for oestrogen receptor‐α (PPT, 100 nM), oestrogen receptor‐β (DPN, 1 nM), or vehicle (ethanol, Control). Both PPT and DPN rapidly decreased left ventricular contractility as demonstrated by the decline in contractile function parameters at 5 min treatment. Left ventricular contractile parameters remained below control values until the end of treatment at 15 min. Note: LVESP, left ventricular end‐systolic pressure; LVDP, left ventricular developed pressure; d*P*/d*t*_max_, maximum rate of ventricular contraction; d*P*/d*t*_min_, maximum rate of ventricular relaxation; VCD 60 D, 60 days after VCD injections; VCD 120 D, 120 days after VCD injections. N = 7 in each treatment group. *P < 0.05 vs intact control at same timepoint

Hearts from ovary intact mice were perfused for 15 minutes with oestrogen receptor‐α (PPT) or ‐β (DPN) agonists. Both PPT and DPN treatment reduced left ventricular systolic pressure at 5 minutes, and systolic pressure remained below intact control values at 10 and 15 minutes of treatment (Figure 4). Rates of contractility (d*P*/d*t*_max_) and relaxation (d*P*/d*t*_min_) were also reduced by oestrogen receptor agonists with the exception of d*P*/d*t*_min_ in 5 minutes DPN‐treated hearts (*P* = 0.081). These data represent the first report showing a rapid decrease in left ventricular contractility with both oestrogen receptor‐α and ‐β activation.

### 3.4 Ovarian function disruption alters oestrogen receptor‐dependent regulation of left ventricular function

**Figure 5:**
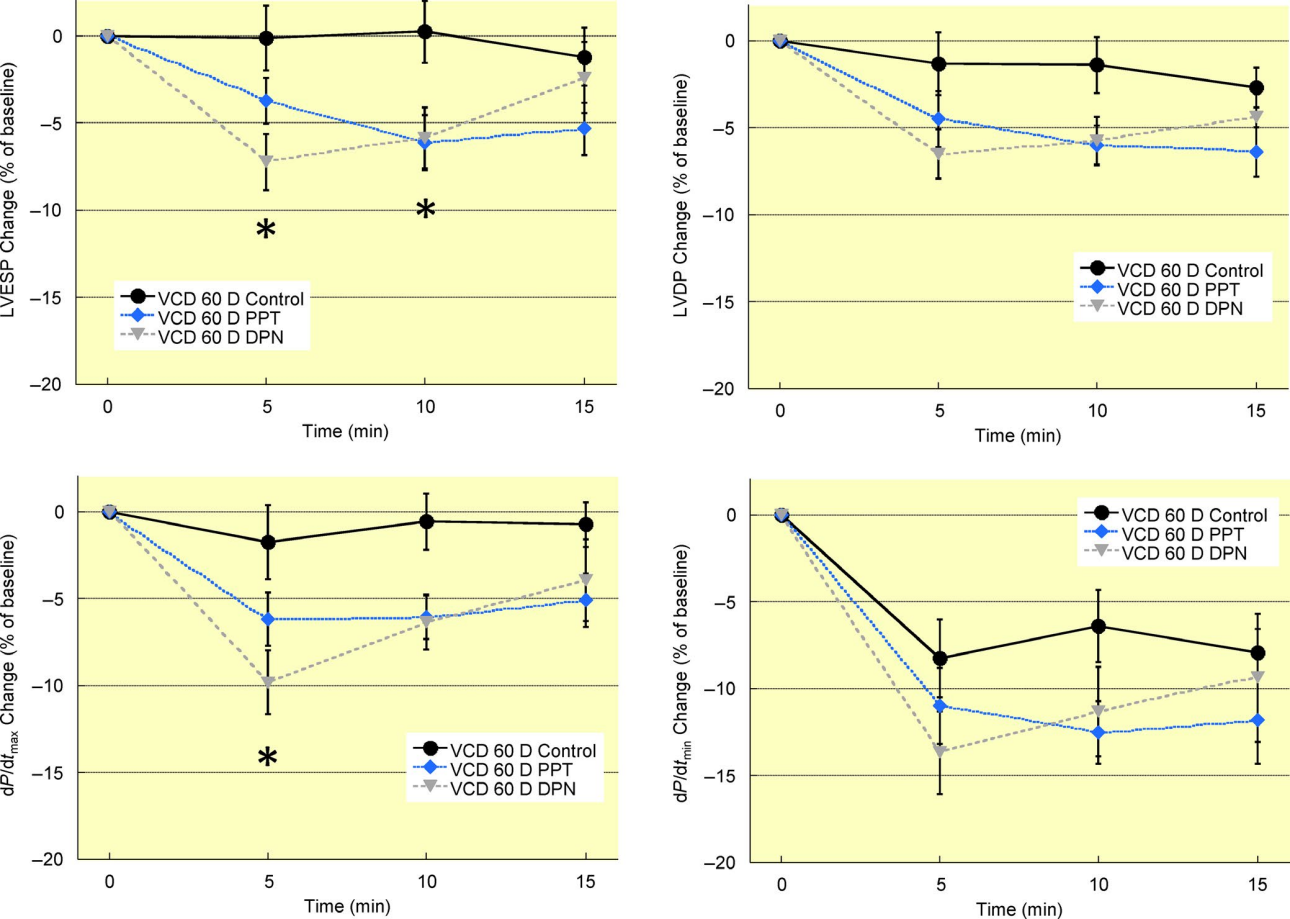
Peri‐menopause impairs control of left ventricular contractility by acute oestrogen receptor activation. Mice were injected with VCD daily for 15 days to induce ovarian failure. 60 days after injections ovarian function is disrupted. Oestrogen receptor‐α (PPT, 100 nM) activation produced a transient decrease in left ventricular systolic pressure and rate of contraction (d*P*/d*t*_max_) that was lost by 15 min of treatment. Oestrogen receptor‐β (DPN, 1 nM) activation did not significantly impact any functional parameter in mice 60 days post‐VCD treatment. Note: LVESP, left ventricular end‐systolic pressure; LVDP, left ventricular developed pressure; d*P*/d*t*_max_, maximum rate of ventricular contraction; d*P*/d*t*_min_, maximum rate of ventricular relaxation; VCD 60 D, 60 days after VCD injections. N = 7 in each treatment group. *P < 0.05 vs VCD 60 days control at same timepoint

**Figure 6:**
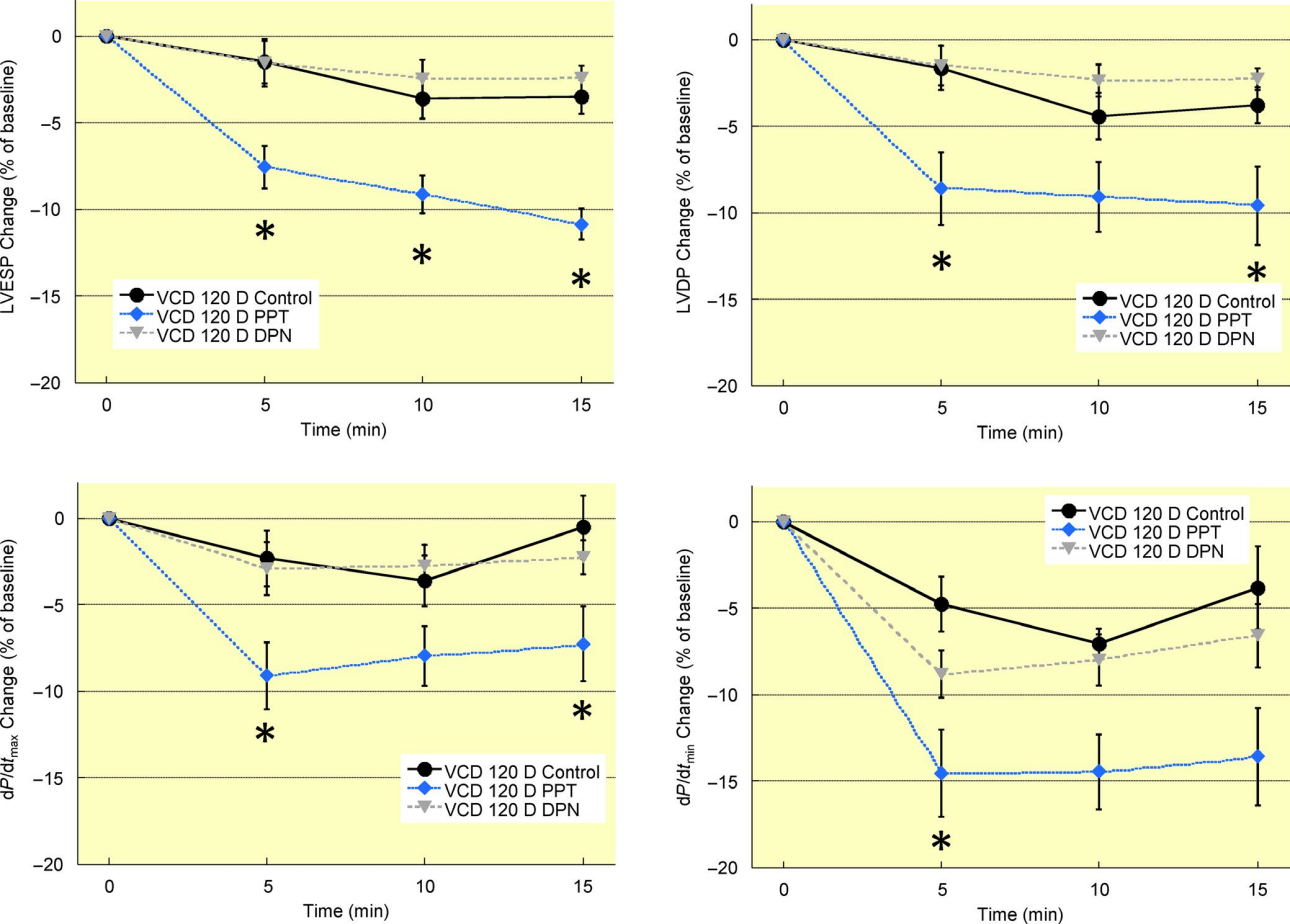
Acute oestrogen receptor‐dependent regulation of left ventricular contractility is partially disrupted by ovarian failures. Hearts from mice 120 days post‐VCD treatment were perfused for 15 min with agonists for oestrogen receptor‐α (PPT, 100 nM), oestrogen receptor‐β (DPN, 1 nM), or vehicle (ethanol, Control). PPT decreased several parameters of left ventricular function up to 15 min. DPN had no detectable effects on left ventricular function. Note: LVESP, left ventricular end‐systolic pressure; LVDP, left ventricular developed pressure; d*P*/d*t*_max_, maximum rate of ventricular contraction; d*P*/d*t*_min_, maximum rate of ventricular relaxation; VCD 120 D, 120 days after VCD injections. N = 7 in each treatment group. *P < 0.05 vs VCD 120 days control at same timepoint

Hearts from mice 60 days post‐VCD treatment had an attenuated response to oestrogen receptor activation (Figure 5). Oestrogen receptor‐β stimulation with DPN transiently decreased contractility as left ventricular end‐systolic pressure was reduced following 5 minutes of treatment. At 10 minutes, the decrease did not reach statistical significance (*P* = 0.0501) nor was the 15 minutes group different from vehicle‐treated controls. Similarly, the decrease in d*P*/d*t*_max_ was significant at 5 minutes but not at 10 or 15 minutes. Oestrogen receptor‐α stimulation reduced left ventricular end‐systolic pressure only at 10 minutes and no other measured parameter was significantly impacted by treatment. In hearts from mice 120 days post‐VCD treatment the depressive effects of oestrogen receptor‐α activation were largely restored (Figure 6). PPT treatment decreased left ventricular end‐systolic pressure at all timepoints examined, and d*P*/d*t*_max_ and d*P*/d*t*_min_ were reduced at some times. Hearts from 120 day VCD mice had no significant response to oestrogen receptor‐β activation. These data are the first to show that the myocardial response to oestrogen receptor activation is affected in a non‐linear fashion by peri‐menopausal ovarian failure.

### 3.5 Oestrogen receptor‐dependent regulation of cardiac myofilament function is affected by ovarian function

**Figure 7:**
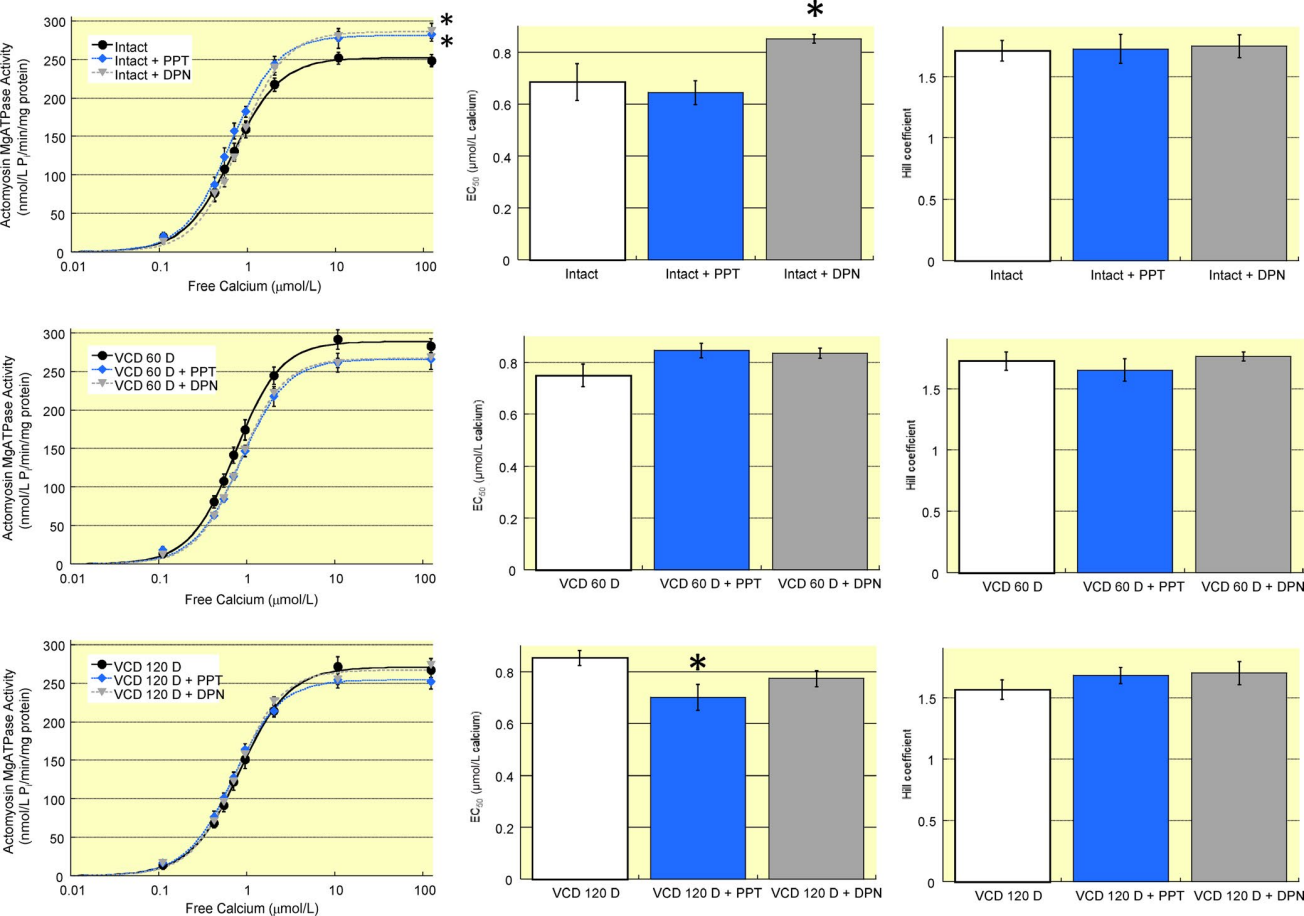
Acute oestrogen receptor‐dependent regulation of cardiac myofilament function is dependent on ovarian function. A, Oestrogen receptor‐α or ‐β stimulation of intact hearts increased maximum actomyosin MgATPase activity as compared to vehicle‐treated controls. B, EC_50_ increased in myofilaments from intact hearts following DPN treatment, but was not altered by PPT. C, Hill coefficient was not altered by oestrogen receptor activation in intact hearts. D, Myofilament function in hearts from mice 60 days after VCD treatment did not show any change in maximum activity in response to oestrogen receptor activation. E, EC_50_ was unaffected by oestrogen receptor activation in 60 day post‐VCD‐treated mouse hearts. F, Hill coefficient was not affected by oestrogen receptor agonists. G, At 120 days after VCD treatment maximum actomyosin MgATPase activity remained unaffected by oestrogen receptor stimulation. H, Oestrogen receptor‐α stimulation with PPT decreased EC_50_ in myofilaments from hearts 120 days post‐VCD injection. I, Hill coefficient was not impacted by either oestrogen receptor agonist. Note: VCD 60 D, 60 days after VCD injections; VCD 120 D, 120 days after VCD injections. N = 7 per group. *P* < 0.05 vs control for each group

Cardiac myofilaments were isolated from hearts treated with oestrogen receptor agonists (15 minutes) or vehicle (15 minutes), and function assessed using an actomyosin MgATPase assay. Myofilaments from hearts of intact mice had an increase in maximum activity from 248 ± 8 nM P_i_/min/mg protein to 282 ± 9 nM P_i_/min/mg protein and 287 ± 10 nM P_i_/min/mg protein following PPT or DPN treatment respectively (Figure 7A). Oestrogen receptor‐β activation with DPN also increased EC_50_ from 0.68 ± 0.07 μM in vehicle‐treated controls to 0.85 ± 0.02 μM (Figure 7B). By day 60 post‐VCD treatment the myofilament response to oestrogen receptor‐α or ‐β activation was abolished (Figure 7D7). At day 120 post‐VCD treatment a myofilament response to PPT treatment was restored. However, in contrast to myofilaments from intact mice, oestrogen receptor‐α activation did not alter maximum actomyosin MgATPase activity (Figure 7G), but it did decrease EC_50_ from 0.85 ± 0.03 μM in vehicle‐treated controls to 0.70 ± 0.05 μM (Figure 7H). These data represent the first investigation of cardiac myofilament changes during a peri‐menopausal phase and reveal a non‐linear change in oestrogen receptor regulation of myofilament function.

### 3.6 Cardiac myofilament protein phosphorylation

**Figure 8:**
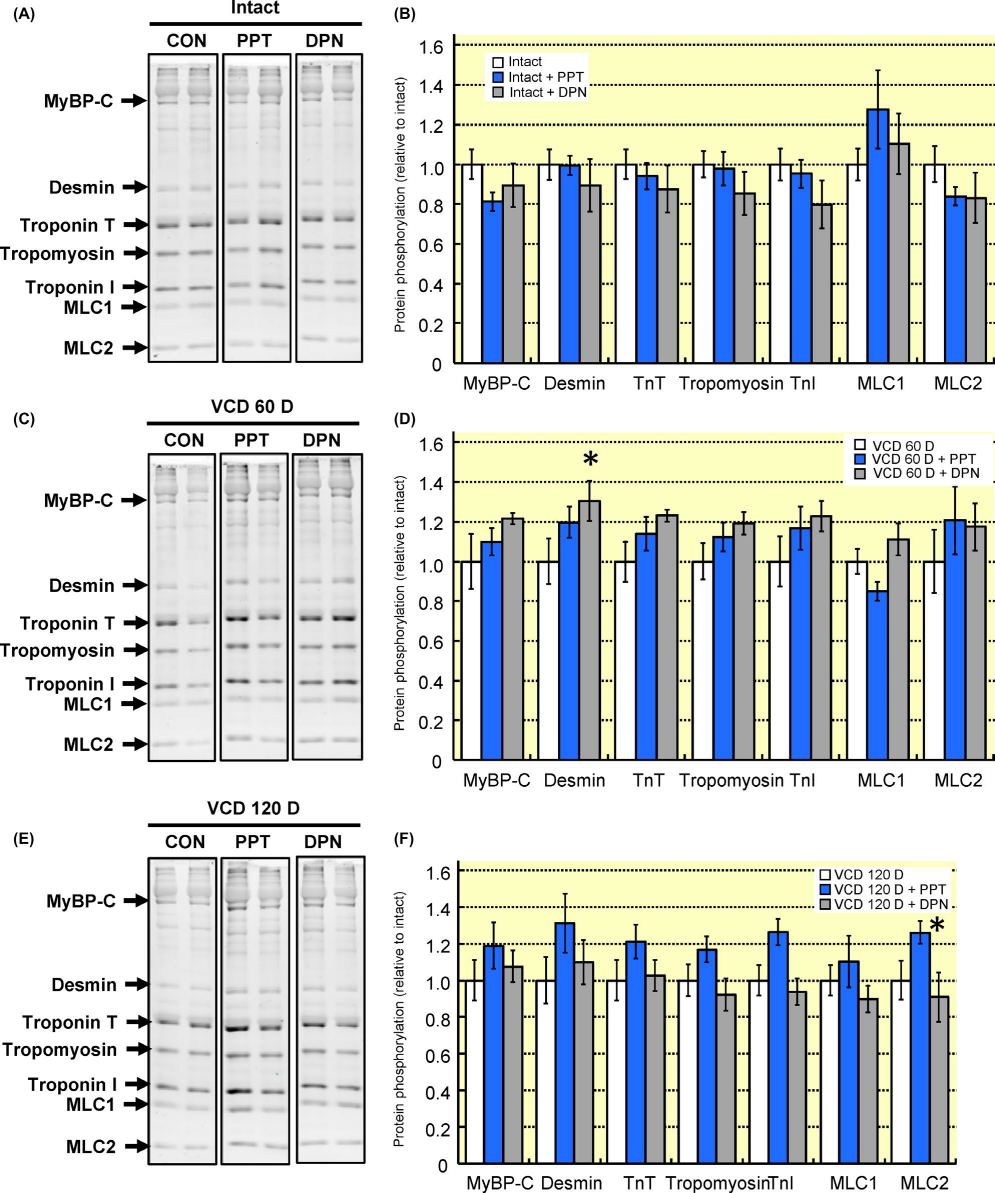
Changes in cardiac myofilament protein phosphorylation following acute oestrogen receptor activation is altered by ovarian function. Hearts were removed from intact mice, 60 days after VCD injections, or 120 days after VCD injections and perfused for 15 min with agonists for oestrogen receptors. A and B, Oestrogen receptor‐α or ‐β stimulation of intact hearts did not impact the total phosphorylation level of any myofilament protein examined as compared to vehicle‐treated controls. C and D, At 60 days following VCD injections desmin phosphorylation was increased with oestrogen receptor‐β stimulation (DPN) and MLC1 phosphorylation was decreased by oestrogen receptor‐α activation (PPT). E and F, At 120 days after VCD injections oestrogen receptor‐α activation (PPT) increased MLC2 phosphorylation. Note: MyBP‐C, myosin binding protein C; TnT, troponin T; TnI, troponin I, MLC1, myosin light chain 1; MLC2, myosin light chain 2; VCD 60 D, 60 days after VCD injections; VCD 120 D, 120 days after VCD injections. N = 7 per group. *P < 0.05 vs vehicle‐treated control from same group

Cardiac myofilaments were isolated from hearts treated with oestrogen receptor agonists or vehicle. In hearts from intact mice neither PPT nor DPN treatment significantly impacted the total phosphorylation level of any measured myofilament protein (Figure 8A and 8). At 60 days post‐VCD treatment, oestrogen receptor‐β stimulation increased total desmin phosphorylation, while oestrogen receptor‐α activation decreased myosin light chain 1 phosphorylation (Figure 8C and 8). At the end of the VCD‐induced peri‐menopausal period myosin light chain 2 phosphorylation was significantly increased by PPT treatment (Figure 8E and 8). These data are consistent with our myofilament actomyosin MgATPase data showing that transition through a peri‐menopausal phase alters the ability of oestrogen receptor activation to regulate cardiac myofilaments.

### 3.7 Cardiac oestrogen receptor‐α and ‐β expression

**Figure 9:**
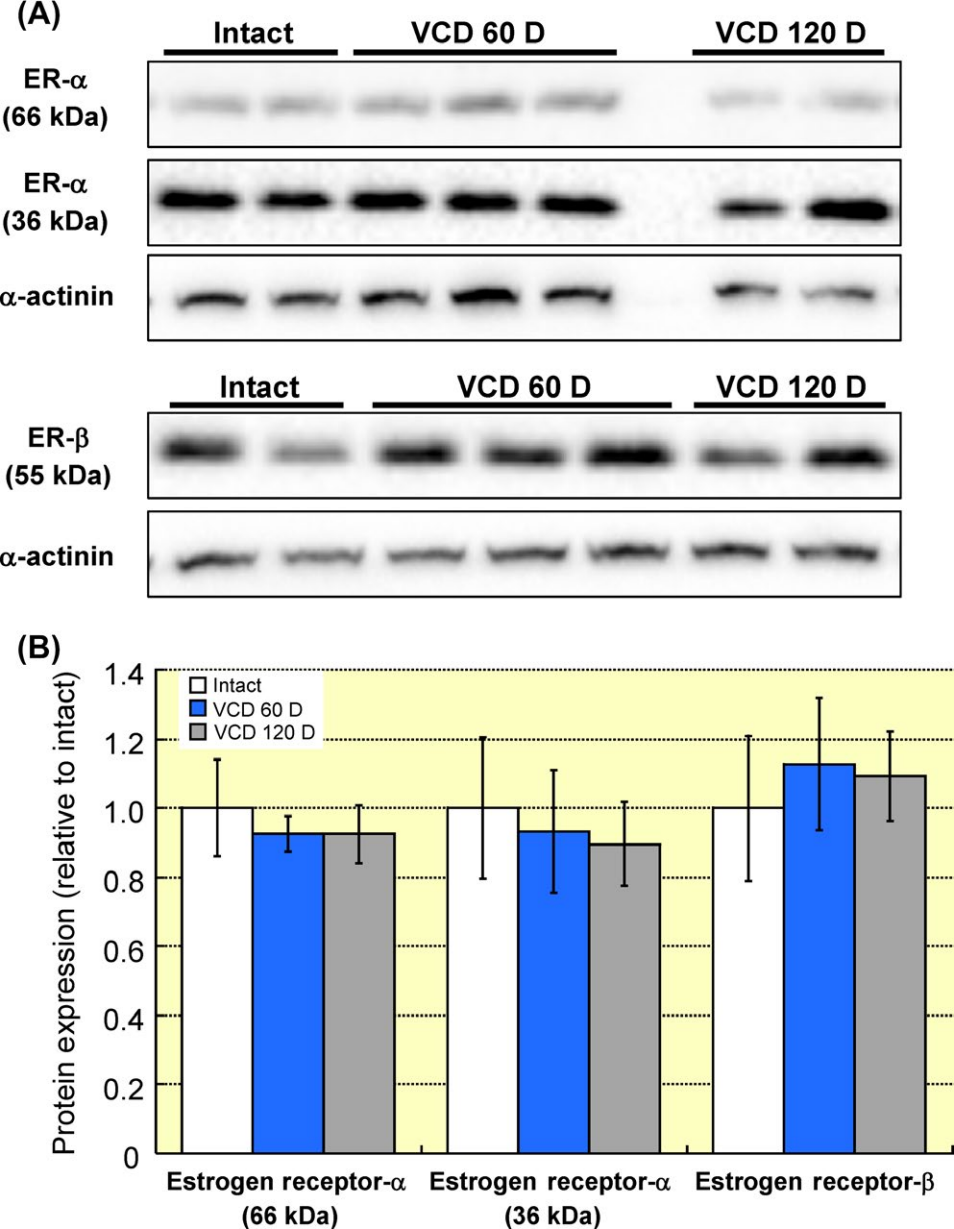
Myocardial oestrogen receptor expression is not affected by VCD‐induced ovarian failure. Myocardial homogenates from intact mice or mice at 60 and 120 days after VCD injection were resolved by SDS‐PAGE and probed for oestrogen receptor‐α and ‐β proteins. Total protein loading was assessed by blotting for α‐actinin. A, Oestrogen receptor‐α immunoblotting revealed bands corresponding to the 66 and 36 kDa isoforms. Protein expression for both splice variants was unaltered during peri‐menopause as compared to intact controls. B, Immunoblotting for oestrogen receptor‐β protein found no differences across groups. Note: ER‐α, oestrogen receptor‐α protein; ER‐β, oestrogen receptor‐β protein; VCD 60 D, 60 days after VCD injections; VCD 120 D, 120 days after VCD injections. N = 4 per group

Since peri‐menopause and ovarian failure are associated with fluctuating—followed by declining—oestrogen levels, we examined whether the cardiac expression of oestrogen receptors changes during the peri‐menopausal transition. Myocardial homogenates were probed with antibodies for oestrogen receptor‐α or ‐β protein. No significant differences were found between hearts from intact mice and mice 60 or 120 days following VCD injections (Figure 9).

## 4 DISCUSSION

This study is the first to investigate changes in cardiac physiology and myocardial regulation that occur during the peri‐menopausal period. We found that myocardial contractile function remained unchanged over the peri‐menopausal period. However, an investigation of myofilament function revealed that actomyosin MgATPase activity varied across time of the menopausal transition, and was associated with changes in myocardial cytokines, decreased Akt activation and a general decrease in myofilament protein phosphorylation. Peri‐menopausal changes in the ability of oestrogen receptors to regulate myocardial contractility and myofilament activation were complex in their non‐linearity and depended on the type of oestrogen receptor. The alterations in oestrogen receptor‐dependent regulation of the heart during peri‐menopause were not driven by changes in receptor protein expression levels. Overall, this study demonstrates that significant and complex cardiac changes occur as a result of fluctuating hormone levels during the peri‐menopaual period and before complete ovarian failure has occurred to start menopause. The molecular changes associated with peri‐menopause do not manifest as overt functional changes at the level of the whole heart, indicating a compensatory state in which the heart adapts at a molecular level to maintain function in an altered hormonal state.

VCD‐induced ovarian failure is a powerful tool to model and investigate the cardiac effects of hormonal changes that occur during peri‐menopause and after the onset of menopause. The vast majority of basic science studies that investigated the effects of menopause on the heart used OVX animals. While these studies provide valuable insight into the effects of oestrogen deficiency on cardiac physiology and disease susceptibility, the abrupt loss of oestrogens does not recapitulate the gradual decline in ovarian function experienced by most women. According to data presented in this study, the peri‐menopausal phase induces a significant molecular remodelling of the heart, which is absent in OVX animals. Moreover, the use of VCD to accelerate follicular atresia leaves intact residual ovarian tissue to secrete androgens. Interestingly, the effects of VCD appear to be confined to ovarian tissue.^25^ This specificity of action enhances the potential of VCD‐induced ovarian failure to act as an effective model to investigate the hormonal changes associated with menopause.

Historically, the effects of steroid hormones were assigned to genomic pathways. More recent studies have uncovered rapid, non‐genomic effects of oestrogen receptor activation in the heart, in particular the rapid cardioprotective effects of oestrogen receptor stimulation against ischemia‐reperfusion injury.^25^ The current study is the first to show that acute activation of cardiac oestrogen receptors rapidly impacts myocardial function and is the only investigation to link physiological changes to the activation of different oestrogen receptor types, namely α and β. We previously reported that oestrogen receptor‐α activation of myocardium mediates myofilament effects within 1 minute of treatment,^26^ but beyond this study the physiological effects of acute oestrogen receptor activation on the heart had not been explored.

Our study presents the novel finding that signalling through ERα and ERβ in the heart is modified during the transition through menopause, without a significant change in oestrogen receptor protein expression levels. The reason for this phenomenon is unknown but other studies offer insight into possible mechanisms. Palmitoylation of ER cysteine residues is critical for receptor anchoring in the cell membrane and positioning close to second messengers.^27^, ^28^ Disruption of ER palmitoylation alters co‐localization with signalling molecules and impacts the acute response to 17β‐estradiol.^29^, ^30^ In additional to palmitoylation other post‐translational modifications of ER include methylation, sumoylation, acetylation, ubiquitination and phosphorylation (reviewed in^31^). Interestingly, some of these post‐translational modifications are influenced by oestrogen levels, providing a mechanism by which changes in oestrogen levels can influence ER signalling. It is feasible that the changes in ER responsiveness during peri‐menopause were the result of oestrogen‐dependent changes in ER post‐translational modifications.

Cardiac myofilaments are the central contractile apparatus of the heart. Changes in protein composition or covalent modifications such as phosphorylation impact myofilament function, which in turn can alter cardiac performance or disease susceptibility. A number of studies examined the effects of OVX on cardiac myofilament function and uncovered effects associated with oestrogen loss.^13^, ^32^, ^33^, ^34^ However, no previous study has investigated the effects of oestrogen loss on myofilament protein phosphorylation, nor has any investigation determined the effects of a gradual oestrogen loss on myofilament function or protein phosphorylation. Data presented in this study show that changes in myofilament activity and protein phosphorylation occur during peri‐menopause and that the alterations do not progress linearly with oestrogen loss. Interestingly, there appears to be a disconnect between changes in cardiac myofilament activity with peri‐menopause and whole heart function. As peri‐menopause progressed to complete ovarian failure, whole heart function remained unaffected, but calcium‐actomyosin MgATPase curves shifted at both 60 days (increased maximum activity) and 120 days (increased EC_50_) post‐VCD treatment. The uncoupling between myofilament function and myocardial contractility suggests that other molecular modifications occur simultaneously with myofilament alterations to create a new homeostasis. Further investigations are required to identify what these changes are.

The mechanism by which ovarian failure impacts cardiac myofilaments are complex and likely involve multiple subcellular elements. We show that myocardial TGF‐β1 increases in peri‐menopause but not at the end of menopause. Kamada et al^21^ reported an increase in serum TGFβ1 early in post‐menopausal women, but this is the first report of myocardial changes. To our knowledge no study has examined the effects of TGF‐β1 on cardiac myofilament activation. However, TGF‐β1 has been linked to Akt inhibition^35^ which we show in the current study. The reduction in Akt activity by itself is expected to decrease myofilament calcium sensitivity^24^ which we did not observe, although we did see a decrease in myofilament phosphorylation consistent with decreased kinase activity. The uncoupling between TGF‐β1, Akt activation, and alterations in myofilament function suggests other simultaneous changes. Romero‐Aleshire et al^36^ showed that ovarian failure induced by VCD leads to insulin resistance and a disruption in energy metabolism. The stress from altered myocardial metabolism—including an increase in reactive oxygen species—negatively impacts myofilament activation^37^ and could explain the depression in myofilament calcium sensitivity. Pollow and colleagues^7^ found that angiotensin II sensitivity was enhanced in a model of VCD‐induced ovarian failure and Palomeque et al^38^ reported that angiotensin II decreases myofilament calcium sensitivity through a p38 MAPK‐dependent pathway. These two studies suggest that the observed decrease in myofilament calcium sensitivity in our study may be mediated by a heightened responsiveness to circulating angiotensin II levels. Overall the numerous and varied signalling cascades and intracellular elements affected directly or indirectly by decreased oestrogen create a complex network of changes that may be invoked to explain differences in myofilament function.

VCD‐induced ovarian failure decreases the synthesis of oestrogen and progesterone, but leaves intact the ability to produce androgens. The effects reported in this study may not simply be a result of decreased oestrogen levels, but instead a more complex outcome from other hormonal changes. Wattanapermpool^39^ treated ovariectomized rats with daily progesterone injections and reported an increase in maximum actomyosin MgATPase activity but no change in calcium sensitivity. Our finding that maximum actomyosin MgATPase activity tended to increase during the peri‐menopausal period when progesterone levels were decreasing is contrary to the results of Wattanapermpool. It should be noted that Wattanapermpool treated rats following ovariectomy (ie, post‐menopausal), whereas the current study looked at the peri‐menopausal period. The impact of testosterone on cardiac myofilaments has not been examined in female animals. Pirompol et al^40^ showed that intact male rats treated with supraphysiological levels of testosterone for up to 12 weeks exhibited a decrease in myofilament activation. More recently, Ayez et al^41^ found that gonadectomy of male mice at 4 weeks of age produced an increase in maximum actomyosin MgATPase activity at 16‐18 months of age. While these studies suggest that the increase in actomyosin MgATPase activity we observed in VCD‐treated mice at 60 days may be due to testosterone, the differences in sex, species and age across all studies make this link tenuous. In short, the lack of studies using chronic progesterone or testosterone treatment in female animals make it difficult to determine which changes we observed in our study may be ascribed to these hormones and underscores the need for further sex‐based research.

Our discovery that myosin binding protein C and troponin I phosphorylation are decreased in cardiac myofilaments from peri‐menopausal mice predicts an increase in myofilament calcium sensitivity,^42^ whereas we found no change. The lack of an expected functional change despite a decrease in myofilament protein phosphorylation levels may be a reflection of the complex and contradictory effects these covalent modifications exert on myofilament activity. For example, while PKA‐dependent phosphorylation of troponin I at serines 23 and 24 decreases myofilament calcium sensitivity, the phosphorylation of threonine 144 in troponin I by PKC increases myofilament calcium sensitivity.^43^ Previous work by Sumandea et al^44^ showed that 3 of the 4 known phosphorylation sites in troponin T actually had no significant impact on myofilament function. Therefore, the functional impact of myofilament protein phosphorylation changes cannot be predicted based on singular alterations, but rather are the product of covalent modifications across the contractile apparatus.

After menopause the risk for CVD in women rises dramatically to equal or even exceed levels seen in age‐matched men. The correlation between the onset of menopause, the resultant decline in circulating oestrogens, and an increase in CVD risk led to the “Oestrogen Protection Hypothesis”. The mechanisms underlying oestrogen protection of the cardiovascular system are complex. ERα stimulation in endothelial cells activates a number of intracellular signalling pathways which ultimately increase nitric oxide production by eNOS.^45^, ^46^ Nitric oxide release by endothelial cells offers protection by inhibiting neointimal hyperplasia and leukocyte accumulation following vascular injury. Endothelial nitric oxide may also mediate vasodilation through interaction with local vascular smooth muscle cells, which themselves are targeted directly by oestrogens. Oestrogen receptor activation in vascular smooth muscle cells activates a PP2A‐Akt cascade to inhibit the cell growth and migration that is essential for atherosclerosis.^47^ Acute activation of oestrogen receptors on cardiac myocytes has repeatedly been shown to protect against ischemia‐reperfusion injury,^48^, ^49^ while ERβ activation mediates anti‐hypertrophic effects by promoting the sequestration of transcription factors away from the nucleus^50^ and by inhibiting activation of the pro‐hypertrophic calcium/calmodulin‐dependent protein kinase II.^51^

The protective potential of oestrogens was initially supported by studies done in non‐human animals and by the Nurses' Health Study (NHS),^3^ but later contradicted by the Women's Health Initiative (WHI)^52^ and the Heart and Oestrogen/Progestin Replacement Study (HERS).^53^ The reasons for the contradictory findings amongst so‐called “hormone replacement therapy” trials are numerous. A review by Miller and Harman^54^ points out that the negative findings of the WHI are actually a misinterpretation in which the results ascribed to a specific oral formulation (0.625 mg conjugated equine oestrogen plus 2.5 mg medroxyprogesterone acetate daily) are inappropriately applied to all oestrogen‐based therapies. They further note conjugated equine oestrogen is a mixture of numerous oestrogens with widely variable formulations. When taken in oral form conjugated equine oestrogens experience a first‐pass effect in the liver where they may increase inflammatory and procoagulant proteins. The differences in hormone replacement therapy formulations and route of delivery have been examined in the ESTHER study,^55^ but additional investigations are required. Miller and Harman^54^ also note that women with pre‐existing metabolic comorbidities including hyperlipidemia and insulin resistance had higher rates of adverse events. Trials in which these women were not analysed separately may obscure or even overwhelm any positive effects that occurred in women without disrupted metabolic profiles.

One of the most commonly raised arguments to explain the discrepant findings of hormone replacement therapies focus on the age, or more accurately the delay to treatment initiation. Post hoc subgroup analysis of some of these clinical studies revealed an interesting trend in which the timing of oestrogen replacement therapy appeared to be important for outcomes and has come to be known as the “Timing Hypothesis”. In general, starting hormone replacement therapy within 10 years of menopause or oophorectomy yielded significantly better outcomes than groups in which treatment onset was delayed beyond a decade.

The premise of the Timing Hypothesis is that the heart changes during and after the transition to menopause, but no study has ever examined temporal changes associated with this transition. Our data clearly show that the myocardial response to oestrogen receptor activation differs throughout the peri‐menopausal phase, and that by the onset of menopause at day 120 post‐VCD injections, cardiac regulation by oestrogen receptor types is different from the pre‐menopausal state. Additionally we find that key elements of the heart, namely cardiac myofilaments, are significantly altered as ovarian function declines in a mouse model of VCD‐induced menopause. Together these findings show a fundamental molecular remodelling and significant alteration in the ability of the heart to respond to oestrogen receptor agonists depending on the stage of ovarian failure, which is consistent with the Timing Hypothesis.

### 4.1 Perspectives and significance

The reintroduction of oestrogens after menopause to recapitulate their hypothesized cardioprotective effects in cycling women presupposes that the heart is able to respond to exogenous oestrogens as it did prior to ovarian failure. This study is the first to show that while the global functioning of the heart in terms of contractile performance is unaltered throughout peri‐menopause, the underlying molecular machinery is significantly affected. Cardiac myofilament function and phosphorylation are changed in a non‐linear mode to produce a fundamentally different contractile system by the end of peri‐menopause. Throughout the peri‐menopausal period the ability of the heart to respond to oestrogen receptor activation is also changed significantly and represents a moving target for therapeutically administered oestrogens that may be provided to maintain the cardioprotective phenotype enjoyed by cycling females. Together these findings uncover significant physiological changes that occur in the heart during the peri‐menopausal transition period and underscore the importance of considering timing for hormone replacement therapy.

## 5 NEW AND NOTEWORTHY

Endogenous oestrogens protect females against cardiovascular disease, however, oestrogen replacement therapy is not clearly beneficial. This discrepancy may be explained by the timing of oestrogen therapy in which early intervention is more effective. This study is the first to identify temporal changes in a mouse model of peri‐menopause, including disruptions in cardiac regulation by oestrogen receptors and myofilament function. These results show a complex remodelling during peri‐menopause which yields a fundamentally different heart after menopause.

## CONFLICTS OF INTEREST

The authors declare no conflict of interest.
